# Mental health, cancer risk, and the mediating role of lifestyle factors in the CARTaGENE cohort study

**DOI:** 10.1371/journal.pone.0281588

**Published:** 2023-02-14

**Authors:** Kaitlyn Gilham, Anne Gadermann, Trevor Dummer, Rachel A. Murphy

**Affiliations:** 1 School of Population and Public Health, The University of British Columbia, Vancouver, British Columbia, Canada; 2 Human Early Learning Partnership, School of Population and Public Health, The University of British Columbia, Vancouver, British Columbia, Canada; 3 Centre for Health Evaluation and Outcome Sciences, Providence Health Care Research Institute, Vancouver, British Columbia, Canada; 4 Cancer Control Research, BC Cancer, Vancouver, British Columbia, Canada; University of Taipei, TAIWAN

## Abstract

**Background:**

Evidence on the association between mental health disorders and cancer risk is inconclusive, despite well-established associations between mental health disorders and lifestyle factors such as smoking. This study examines the relationships between depression, anxiety and cancer risk, and the potential mediating effects of lifestyle factors.

**Methods:**

A study of 34,571 participants aged 40–69 years in the CARTaGENE cohort was conducted. Depression was defined by questionnaire (PHQ-9), antidepressant use, and a composite of questionnaire, antidepressant use, or lifetime self-reported physician diagnosis. Anxiety was defined by questionnaire (GAD-7). Co-morbid depression and anxiety was also assessed. Cox regression models were used to investigate associations between mental health and risk of prostate, lung, and all cancers combined. Mediating effects of lifestyle factors were assessed using Baron and Kenny mediation criteria.

**Results:**

There were positive associations between mental health disorders, all cancers and lung cancer risk, however with the exception of anxiety and lung cancer in women (Hazard Ratio [HR] = 1.67, 95% CI: 1.01–2.76), associations were attenuated with adjustment for sociodemographics, health status and lifestyle factors. In the mediation analysis, smoking accounted for 27%, 18%, and 26%, of the total effect between depression (PHQ-9), anxiety, and co-morbidity and lung cancer, respectively in women. In men, smoking accounted for 17% of the total effect between depression (PHQ-9, antidepressant, or lifetime self-report of physician diagnosis) and all cancers.

**Conclusions:**

Positive associations were observed between mental health disorders, all cancer and lung cancer risk, however most relationships were attenuated with adjustment for lifestyle factors. Smoking status mediated a significant proportion of the relationships between mental health disorders and cancer risk.

## Introduction

It is estimated that approximately 1 in 2 Canadians will develop cancer in their lifetime, and 1 in 4 Canadians will die from cancer [1]. Cancer is also the leading cause of death in Canada, responsible for approximately 30% of all deaths [2]. The financial and emotional cost of cancer in Canada is substantial–impacting patients, their caregivers, and the healthcare system. Preventive interventions are crucial to minimize the economic and social impact of the burden of cancer in Canada.

Mental health disorders also have a high public health burden. It is estimated that 1 in 5 Canadians are affected annually by mental health disorders [3]. According to Pearson et al., 11.3% of Canadian adults have met the criteria for a depression disorder at some point in their lifetime, and 8.7% have met the criteria for generalized anxiety disorder (GAD) [4]. Individuals with mental health disorders have been shown to have an increased risk of several chronic illnesses, including cardiovascular disease and diabetes [5–7]. However, evidence on the association between cancer and mental health disorders is largely inconclusive. One meta-analysis of nine studies that assessed the relationship between depression and risk of all cancers reported high heterogeneity across studies and inadequate consideration of covariates such as smoking [8]. Another systematic review and meta-analysis of 25 studies with a pooled sample of 1,469,179 participants and 89,716 incident cases of cancer assessed the association between depression and incident cancer risk [9]. This study found that depression was significantly associated with an increased risk of all cancer, liver, and lung cancer [9]. However, no associations were found for breast, prostate, or colorectal/colon cancer [9]. The review also noted that very few studies adjusted for lifestyle risk factors, such as diet, physical activity, tobacco, or alcohol consumption.

Lifestyle factors are closely associated with anxiety, depression, and cancer. Individuals with depression and anxiety are more likely to smoke, consume higher levels of alcohol, engage in lower levels of physical activity, have lower diet quality, and experience disturbed sleep compared to those without depression or anxiety [10–18]. These lifestyle factors are key components of cancer prevention guidelines [19]. In 2015, an estimated 33% of cancer cases diagnosed were attributable to lifestyle factors; tobacco smoking, low fruit and vegetable intake, physical inactivity, and excess weight [20].

Given the established association between healthy lifestyle behaviours with cancer, and mental health disorders, it is possible that the inconsistent associations between depression, anxiety and cancer risk are due to lack of consideration of the impact of behavioural factors. It may also be pertinent to consider the potential modifying role of sex on relationships. There are well established differences in the prevalence of mental health disorders, symptoms and usage of mental health services [21, 22]. The incidence of many of the most common cancers such as colorectal and lung cancer differ by sex [23, 24], as does engagement in healthy lifestyle behaviours [25]. To meet these gaps, this study aimed to examine associations between mental health disorders and cancer risk. Overall cancer risk was assessed, in addition to prostate cancer and lung cancer, the two most common cancer types among men [26], and the second most common type of cancer among women (lung). Models were also tested for effect modification by sex. The secondary aim was to explore whether lifestyle factors mediated associations between mental health disorders and cancer risk.

## Methods

### Study sample

Participants for the present study were drawn from the CARTaGENE cohort, a study of 43,000 men and women aged 40–69 in Quebec, Canada. Participants were enrolled between 2009 and 2015 as part of the national Canadian Partnership for Tomorrow’s Health (CanPath), a prospective, longitudinal cohort study [27]. Details of the survey methods and cohort profile have previously been published [28]. Ethics approval for this project was obtained from the University of British Columbia Clinical Research Board (Certificate #H17-02706). All participants provided written, informed consent.

At study baseline participants completed self-report questionnaires that asked about sociodemographic factors, medications, and health and lifestyle factors [28]. A proportion of participants attended in-person assessments, which included measurement of height, and bio-impedance measurement of weight. Incident cancers were determined from the provincial cancer registry until 2010. After 2010, incident cancers were defined from hospitalization and medication data collected as part of the administrative health database, Régie de l’assurance maladie du Québec (RAMQ), using Tonelli criteria [29] until April 2018. Tonelli criteria are a set of validated algorithms that can be used to identify chronic conditions from administrative data [29]. The first cancer diagnostic date was used for cases meeting Tonelli criteria.

Participants were excluded if they had any incomplete information from the anxiety (GAD-7) and depression (PHQ-9) questionnaires (n = 2,509), were missing date of cancer diagnosis (n = 34), or reported a prior cancer diagnosis (n = 5,687). Participants who were diagnosed with cancer within the first six months of follow-up (n = 236) were also excluded to minimize reverse causation. After exclusion criteria was applied, the final analytical sample size was 34,571 participants. This included 2,888 total incident cancer cases, with 329 prostate and 305 lung cancer cases.

### Depression

Depression at baseline was assessed using multiple definitions that capture different time frames and severity of depression. Depressive symptoms at baseline were assessed using the validated PHQ-9, a nine-item questionnaire that assesses symptoms of depression over the past two weeks [30]. A score of 10 or higher was used to identify depression [31]. This score has a has a sensitivity of 88% and a specificity of 88% for detecting major depressive disorder in an adult primary care sample [31]. In addition, PHQ-9 scores were considered in statistical models as a continuous variable (S1-S6 Tables in S1 File). Participants self-reported medication use in the CARTaGENE data was organized according to the Anatomical Therapeutic Chemical (ATC) Classification system developed by the WHO [32]. Antidepressant use was defined as current use at baseline of a drug belonging to ATC code N06A [33].

Self-reported depression data was also collected in the baseline questionnaire. Participants were asked “has a physician ever told you that you have depression?” Positive responses to this question were recorded as a lifetime prevalence of depression. Self-reported depression was considered in a composite measure of depression which was a positive response for any of PHQ-9 score ≥10, use of antidepressants, or self-report of physician diagnosis.

### Anxiety

Anxiety symptoms were assessed at baseline using the validated GAD-7, a seven-item self-report scale that assesses symptoms over the past two weeks [34]. A score ≥10 was used to indicate anxiety, which has been shown to have a sensitivity of 89% and specificity of 82% in an adult primary care setting [34]. Continuous GAD-7 scores were also evaluated in statistical models (S1-S6 Tables in S1 File). The variable of self-reported lifetime diagnosis of anxiety by a physician was only collected in the second phase of the CARTaGENE study, resulting in a high proportion of missing data. Thus, anxiety in this study was only assessed using GAD-7.

### Co-morbid depression and anxiety

Co-morbid depression and anxiety was defined as participants who scored a 10 or higher on both the PHQ-9 and the GAD-7 questionnaires.

### Covariates

Covariates assessed for potential confounding and mediating effects were selected based on previous research and included sociodemographic factors (age, sex, ethnicity, education, marital status, and income), lifestyle factors (smoking status, alcohol consumption, sleep, physical activity, body mass index (BMI), fruit and vegetable consumption (servings/day), and health status (previous diagnosis of diabetes [35] or myocardial infarction [36]). In addition, cancer specific risk factors were evaluated. For lung cancer, chronic obstructive pulmonary disorder (COPD) and family history of lung cancer, and for prostate cancer, family history of prostate cancer. Covariates were categorized to align with established thresholds, as outlined below, or according to categories previously defined in analyses of CanPath data [37, 38].

Age was analyzed as a continuous variable. Ethnicity was dichotomized as white and non-white due to small counts in ethnicities other than white. Education was categorized as high school or lower, college, and university or higher. Marital status was dichotomized as living with partners (married or common-law), and without partners (single, divorced, or widowed). Household income was categorized as less than $50,000, $50,000–74,999, $75,000–150,000, and greater than $150,000.

Current smokers were those who had smoked at least 100 cigarettes in their lifetime and smoked in the past 30 days. If participants had smoked at least 100 cigarettes in their lifetime, but not in the past 30 days, they were categorized as past smokers. All other participants were categorized as non-smokers. Participants were classified as abstainers (never drinking alcohol), former (drank alcohol before but not over the past 12 months), occasional (≤2–3 times/month), regular (≥ once/week but ≤2–3 times/week), and habitual drinkers (≥4–5 times/week). Average hours of sleep were categorized as <7 hours, 7–9 hours, and 9+ hours per night [39]. Physical activity was evaluated using the International Physical Activity Questionnaire (IPAQ), which asked about the number of days and usual time spent doing vigorous and moderate activity, walking and sitting in the prior 7 days. Physical activity was subsequently categorized as low, moderate, or high as per IPAQ scoring protocol [40]. BMI was categorized based on standard classification: <25 kg/m^2^ as underweight/normal, 25.0 to 29.9 kg/m^2^ as overweight, and ≥ 30 kg/m^2^ as obese. BMI from measured height/weight was used when available, otherwise self-reported height/weight was used. Servings of fruits and vegetables per day were dichotomized into: <five or ≥ five in line with recommendations for consumption to support overall health [41]. Self-reported diagnosis of diabetes, myocardial infarction, and COPD were recorded as “yes” or “no”. Participants were asked to indicate whether immediate blood relatives (mother, father, children, full and half brothers and sisters) had ever been diagnosed with cancer. If they answered ‘yes’, the type(s) of cancer were queried for each relative.

### Statistical analysis

Missing covariate data was imputed with the *‘Multivariate Imputations by Chained Equation (MICE)’* package in R [42]. The proportion of missing was 0.02% for age, 0.37% for education, 8.36% for income, 1.64 for ethnicity, 0.30% for smoking status, 2.69% for alcohol consumption, 7.21% for physical activity, 13.8% for BMI, 0.27% for sleep, 1.30% for fruit and vegetable consumption, 0.34% for myocardial infarction, 0.45% for diabetes, and 0.46% for COPD, respectively. Sex and self-perceived health were included in the multiple imputation as auxiliary variables; variables correlated with the missing values that increase the likelihood the missing at random assumption holds. Ten iterations and 20 imputations were needed to reach convergence.

Cox proportional hazards regression analysis was used to estimate hazard ratios (HR) and 95% confidence intervals (CIs) between the exposure and outcome variables. The proportional hazards assumption was verified using Schoenfeld residuals with the *‘survival’* package in R [43]. Days of follow-up was used as the time-scale (continuous in days) included as a covariate in the model [44]. Days of follow-up was determined from the difference between first hospitalization or first claim record in the case of Tonelli criteria or date of diagnosis from the cancer registry and date of consent for the baseline questionnaire. Follow-up for non-cases was determined as the difference between date of last linkage to the RAMQ database, or date of death, and date of consent for the baseline questionnaire. The change in coefficient method was used to determine significant confounders with a stepwise procedure, with a significance level of 10%. Models (1 through 3) were progressively adjusted for sociodemographic and health factors. Effect modification was evaluated by adding an interaction term between two variables in a given model. Sex was identified as an effect modifier for all measures of depression and for anxiety, and thus, models were stratified. Analyses were also performed with estimates pooled for men and women for overall cancer and lung cancer (S2, S3, S6 and S7 Tables in S1 File).

In order to clarify the role of lifestyle factors on the causal pathway between mental health disorders and cancer risk, a mediation analysis was performed to calculate the direct, indirect, and total effect using the *mediation* package in R software [45]. First, potential mediating effects were screened to establish relevant exposures, mediators, and outcomes using the Baron and Kenny mediation criteria, which is the most widely used method to assess the mediation [46, 47]. For relationships that met all Baron and Kenny criteria, confidence intervals were estimated using the quasi-Bayesian Monte Carlo method with 1000 simulations [48]. The presence of exposure-mediator interactions within models were also determined to inform inferences about mediation [49]. A p-value below 0.05 (two-sided) was considered as statistically significant.

## Results

A greater prevalence of high PHQ-9 scores, meeting thresholds for depression were observed in female participants, participants with an annual household income of less than $50,000, participants with a high school education or lower, participants who were single, divorced, or widowed, participants with diabetes, and participants with COPD (**Table 1**).

**Table 1:** Descriptive characteristics of the CARTaGENE sample with comparison of PHQ-9 scores.

	Depression status		Overall Analytic Sample
	No (PHQ-9 score <10)	Yes (PHQ-9 score ≥10)	
	n = 32,652 (94.4%)	n = 1,919 (5.6%)	n = 34,571
**Age, Mean (SD)**	53.2 (7.91)	52.0 (7.23)	53.1 (7.88)
**Sex**			
Female	17474 (53.5%)	1178 (61.4%)	18652 (54.0%)
Male	15178 (46.5%)	741 (38.6%)	15919 (46.0%)
**Ethnicity**			
White	29218 (89.5%)	1642 (85.6%)	30860 (89.3%)
Non-white	3434 (10.5%)	277 (14.4%)	3711 (10.7%)
**Income**			
<$50,000	9438 (28.9%)	982 (51.2%)	10420 (30.1%)
$50,000–74,999	7091 (21.7%)	358 (18.7%)	7449 (21.5%)
$75,000–100,000	5188 (15.9%)	230 (12.0%)	5418 (15.7%)
>$100,000	10935 (33.5%)	349 (18.2%)	11284 (32.6%)
**Education**			
High school or lower	6934 (21.2%)	627 (32.7%)	7561 (21.9%)
College	10662 (32.7%)	632 (32.9%)	11294 (32.7%)
University	15056 (46.1%)	660 (34.4%)	15716 (45.5%)
**Marital status**			
Married	22797 (69.8%)	991 (51.6%)	23788 (68.8%)
Single, divorced, or widowed	9855 (30.2%)	928 (48.4%)	10783 (31.2%)
**Diabetes**			
Yes	1967 (6.0%)	223 (11.6%)	2190 (6.3%)
No	30685 (94.0%)	1696 (88.4%)	32381 (93.7%)
**Myocardial infarction**			
Yes	661 (2.0%)	46 (2.4%)	707 (2.0%)
No	31991 (98.0%)	1873 (97.6%)	33864 (98.0%)
**COPD**			
Yes	820 (2.5%)	165 (8.6%)	985 (2.8%)
No	31832 (97.5%)	1754 (91.4%)	33586 (97.2%)
**Family cancer history**			
Yes	18135 (55.5%)	1083 (56.4%)	19218 (55.6%)
No	14517 (44.5%)	836 (43.6%)	15353 (44.4%)
**Smoking status**			
Current smoker	5409 (16.6%)	601 (31.3%)	6010 (17.4%)
Past smoker	12950 (39.7%)	659 (34.3%)	13609 (39.4%)
Never smoked	14293 (43.8%)	659 (34.3%)	14952 (43.3%)
**Alcohol consumption**			
Abstainer	1727 (5.3%)	132 (6.9%)	1859 (5.4%)
Former drinker	2883 (8.8%)	327 (17.0%)	3210 (9.3%)
Occasional drinker	8024 (24.6%)	628 (32.7%)	8652 (25.0%)
Regular drinker	12437 (38.1%)	484 (25.2%)	12921 (37.4%)
Habitual drinker	7581 (23.2%)	348 (18.1%)	7929 (22.9%)
**Physical activity**			
Low	6569 (20.1%)	592 (30.8%)	7161 (20.7%)
Moderate	11766 (36.0%)	682 (35.5%)	12448 (36.0%)
High	14317 (43.8%)	645 (33.6%)	14962 (43.3%)
**BMI**			
Underweight	242 (0.7%)	18.0 (0.9%)	260 (0.8%)
Normal	10205 (31.3%)	436 (22.7%)	10641 (30.8%)
Overweight	14021 (42.9%)	738 (38.5%)	14759 (42.7%)
Obese	8184 (25.1%)	727 (37.9%)	8911 (25.8%)
** Fruit & vegetable consumption**			
<5 servings	16061 (49.2%)	1146 (59.7%)	17207 (49.8%)
≥5 servings	16591 (50.8%)	773 (40.3%)	17364 (50.2%)

^**†**^Non-smoker: has never smoked, former smoker: has smoked at least 100 cigarettes before but not within the past 30 days, current smoker: has smoked more than 100 cigarettes in lifetime and has smoked within the last 30 days

^‡^Abstainer: has never drunk alcohol, former: has drunk alcohol before but not over the past 12 months, occasional: drank ≤2–3 times/month over the past 12 months, regular: drank ≥ once/week but ≤2–3 times/week, habitual drinkers: drank ≥ 4–5 times/week.

Similar to the comparison of depression status, greater proportions of GAD-7 scores that met thresholds for anxiety were observed in female participants, participants with an annual household income of less than $50,000, participants with a high school education or lower, participants who were single, divorced, or widowed, participants with type II diabetes, and participants with COPD (**Table 2**).

**Table 2:** Characteristics of the CARTaGENE sample with exclusion criteria applied with comparison of GAD-7 scores.

	Anxiety status		Overall Analytic Sample
	No (GAD-7 score <10)	Yes (GAD-7 score ≥10)	
	n = 32,982 (95.4%)	n = 1,589 (4.6%)	n = 34,571
**Age, Mean (SD)**	53.1 (7.90)	52.3 (7.37)	53.1 (7.88)
**Sex**			
Female	17641 (53.5%)	1011 (63.6%)	18652 (54.0%)
Male	15341 (46.5%)	578 (36.4%)	15919 (46.0%)
**Ethnicity**			
White	29491 (89.4%)	1369 (86.2%)	30860 (89.3%)
Non-white	3491 (10.6%)	220 (13.8%)	3711 (10.7%)
**Income**			
<$50,000	9615 (29.2%)	805 (50.7%)	10420 (30.1%)
$50,000–74,999	7160 (21.7%)	289 (18.2%)	7449 (21.5%)
$75,000–100,000	5211 (15.8%)	207 (13.0%)	5418 (15.7%)
>$100,000	10996 (33.3%)	288 (18.1%)	11284 (32.6%)
**Education**			
High school or lower	6991 (21.2%)	570 (35.9%)	7561 (21.9%)
College	10798 (32.7%)	496 (31.2%)	11294 (32.7%)
University	15193 (46.1%)	523 (32.9%)	15716 (45.5%)
**Marital status**			
Married	22897 (69.4%)	891 (56.1%)	23788 (68.8%)
Single, divorced, or widowed	10085 (30.6%)	698 (43.9%)	10783 (31.2%)
**Diabetes**			
Yes	2033 (6.2%)	157 (9.9%)	2190 (6.3%)
No	30949 (93.8%)	1432 (90.1%)	32381 (93.7%)
**Myocardial infarction**			
Yes	670 (2.0%)	37 (2.3%)	707 (2.0%)
No	32312 (98.0%)	1552 (97.7%)	33864 (98.0%)
**COPD**			
Yes	859 (2.6%)	126 (7.9%)	985 (2.8%)
No	32123 (97.4%)	1463 (92.1%)	33586 (97.2%)
**Family history of cancer**			
No	14677 (44.5%)	676 (42.5%)	15353 (44.4%)
Yes	18305 (55.5%)	913 (57.5%)	19218 (55.6%)
**Smoking status**			
Current smoker	5537 (16.8%)	473 (29.8%)	6010 (17.4%)
Past smoker	13039 (39.5%)	570 (35.9%)	13609 (39.4%)
Never smoked	14406 (43.7%)	546 (34.4%)	14952 (43.3%)
** Alcohol consumption**			
Abstainer	1745 (5.3%)	114 (7.2%)	1859 (5.4%)
Former drinker	2976 (9.0%)	234 (14.7%)	3210 (9.3%)
Occasional drinker	8144 (24.7%)	508 (32.0%)	8652 (25.0%)
Regular drinker	12483 (37.8%)	438 (27.6%)	12921 (37.4%)
Habitual drinker	7634 (23.1%)	295 (18.6%)	7929 (22.9%)
** Physical activity**			
Low	6726 (20.4%)	435 (27.4%)	7161 (20.7%)
Moderate	11902 (36.1%)	546 (34.4%)	12448 (36.0%)
High	14354 (43.5%)	608 (38.3%)	14962 (43.3%)
**BMI**			
Underweight	243 (0.7%)	17.0 (1.1%)	260 (0.8%)
Normal	10212 (31.0%)	429 (27.0%)	10641 (30.8%)
Overweight	14136 (42.9%)	623 (39.2%)	14759 (42.7%)
Obese	8391 (25.4%)	520 (32.7%)	8911 (25.8%)
**Fruit & vegetable consumption**			
<5 servings	16286 (49.4%)	921 (58.0%)	17207 (49.8%)
≥5 servings	16696 (50.6%)	668 (42.0%)	17364 (50.2%)

^**†**^Non-smoker: has never smoked, former smoker: has smoked at least 100 cigarettes before but not within the past 30 days, current smoker: has smoked more than 100 cigarettes in lifetime and has smoked within the last 30 days ^‡^Abstainer: has never drunk alcohol, former: has drunk alcohol before but not over the past 12 months, occasional: drank ≤2–3 times/month over the past 12 months, regular: drank ≥ once/week but ≤2–3 times/week, habitual drinkers: drank ≥ 4–5 times/week.

Mental health characteristics of the analytic sample are presented in **Table 3.** Depression defined by antidepressant use was observed in 9.2% of the analytic sample, and depression defined by PHQ-9 scores was observed in 5.6% of the sample. Self-reported lifetime depression diagnosis by a physician was observed for a higher proportion of the sample (21.5%). Anxiety defined by GAD-7 scores occurred in 4.6% of the sample, and comorbid anxiety and depression was observed in 2.7%.

**Table 3:** Mental health characteristics of the CARTaGENE sample by sex at baseline.

	Women	Men	Overall	p-value
	n = 18,652	n = 15,919	n = 34,571	
**GAD-7 score**				<0.001
Mean (SD)	2.8 (3.6)	2.2 (3.2)	2.5 (3.4)	
10^th^ percentile	0	0	0	
90^th^ percentile	7	6	6	
**PHQ-9 score**				<0.001
Mean (SD)	3.2 (3.7)	2.6 (3.5)	2.9 (3.6)	
10^th^ percentile	0	0	0	
90^th^ percentile	8	7	7	
**Anxiety (GAD score ≥10)**				<0.001
Yes	1011 (5.4%)	578 (3.6%)	1589 (4.6%)	
No	17641 (94.6%)	15341 (96.4%)	32982 (95.4%)	
**Depression (PHQ score ≥10)**				<0.001
Yes	1178 (6.3%)	741 (4.7%)	1919 (5.6%)	
No	17474 (93.7%)	15178 (95.3%)	32652 (94.4%)	
**Depression diagnosis by physician**				<0.001
Yes	4858 (26.0%)	2561 (16.1%)	7419 (21.5%)	
No	13794 (74.0%)	13358 (83.9%)	27152 (78.5%)	
**Antidepressant use**				
Yes	2271 (12.2%)	920 (5.8%)	3191 (9.2%)	<0.001
No	16381 (87.8%)	14999 (94.2%)	31380 (90.8%)	
**Depression (PHQ-9, antidepressant use, or physician diagnosis)**				<0.001
Yes	5975 (32.0%)	3168 (19.9%)	9143 (26.4%)	
No	12677 (68.0%)	12751 (80.1%)	25428 (73.6%)	
**Comorbid depression and anxiety (GAD and PHQ-9 scores ≥10)**				<0.001
Yes	599 (3.2%)	339 (2.1%)	938 (2.7%)	
No	18053 (96.8%)	15580 (97.9%)	33633 (97.3%)	

Women had a higher mean GAD-7 score (2.8) and PHQ-9 score (3.2) compared to men (2.2 and 2.6, respectively) (**Table 3**). The proportion of women with a GAD-7 score over the threshold of 10 points indicating anxiety (5.4%) was also higher than men (3.6%) (**Table 3**). This disparity was also reflected in the proportion of women with depression (6.3%, compared to 4.7% for men), the proportion of women diagnosed with depression by a physician in their lifetime (26.0%, compared to 16.1% for men), proportion of women using antidepressants (12.2%, compared to 5.8% for men), and proportion of women with comorbid depression and anxiety (3.2%, compared to 2.1% for men) (**Table 3**).

### Depression, anxiety, and risk of cancer

The median (min, max) follow-up time for all cancers was 3.5 years (182 days, 8.5 years) for men and 3.4 years (181 days, 8.4 years) for women. Among all three depression definitions, the risk estimates for men and women tended to be above 1.0, although, generally associations were statistically non-significant (**Table 4**). Similarly, no associations were observed between PHQ-9 scores on a continuous scale and risk of cancer (S1, S2 Tables in S1 File). The composite measure of depression was the only notable exception, but risk estimates were attenuated in Model 3 with additional adjustment for lifestyle behaviours. Anxiety was not associated with cancer risk in men or women (Table 4 and S1 Table in S1 File). Risk estimates for women tended to be higher than those for men for anxiety (GAD-7), depression (PHQ-9), and comorbid anxiety and depression. Estimates however, did not reach statistical significance with the exception of Model 1 for comorbid anxiety and depression among women.

**Table 4:** Hazard ratios between mental health disorders and subsequent diagnosis of all cancers, stratified by sex.

	No. of Events/No. of Participants	Person-years	Model 1† HR (95% CI)	Model 2‡ HR (95% CI)	Model 3§ HR (95% CI)
**Depression (PHQ-9)**					
Women					
No	1419/17474	101460	Ref	Ref	Ref
Yes	108/1178	7045	1.14 (0.94–1.39)	1.13 (0.93–1.38)	1.08 (0.88–1.32)
Men					
No	1301/15178	91312	Ref	Ref	Ref
Yes	60/741	4570	1.05 (0.81–1.36)	1.06 (0.81–1.37)	0.99 (0.76–1.29)
**Depression (Antidepressant use)**					
Women					
No	1346/16381	95983	Ref	Ref	Ref
Yes	181/2271	12522	1.04 (0.89–1.21)	1.03 (0.88–1.20)	1.00 (0.85–1.17)
Men					
No	1280/14999	90627	Ref	Ref	Ref
Yes	81/920	5255	1.11 (0.89–1.39)	1.11 (0.89–1.39)	1.07 (0.85–1.34)
**Depression (PHQ-9, antidepressant use, or self-report of physician diagnosis)**					
Women					
No	1011/12677	73382	Ref	Ref	Ref
Yes	513/5975	35122	1.06 (0.95–1.18)	1.05 (0.95–1.17)	1.01 (0.91–1.13)
Men					
No	1057/12751	76477	Ref	Ref	Ref
Yes	303/3168	19404	**1.15 (1.01–1.31)**	**1.14 (1.01–1.28)**	1.11 (0.97–1.26)
**Anxiety (GAD-7)**					
Women					
No	1431/17641	95983	Ref	Ref	Ref
Yes	96/1011	12522	1.12 (0.91–1.38)	1.12 (0.91–1.38)	1.08 (0.88–1.34)
Men					
No	1313/15341	90627	Ref	Ref	Ref
Yes	48/578	5255	1.05 (0.79–1.41)	1.06 (0.79–1.41)	1.00 (0.75–1.34)
**Comorbid anxiety and depression**					
Women					
No	1463/18053	104804	Ref	Ref	Ref
Yes	64/599	3701	**1.28 (1.00–1.65)**	1.28 (0.99–1.64)	1.21 (0.94–1.56)
Men					
No	1331/15580	93739	Ref	Ref	Ref
Yes	30/339	2143	1.18 (0.82–1.70)	1.18 (0.82–1.70)	1.09 (0.76–1.58)

†Model 1: Adjusted for age (continuous), education, ethnicity, and income

‡Model 2: Adjusted for age (continuous), education, ethnicity, income, myocardial infarction, diabetes, and family history of cancer

**§**Model 3: Adjusted for all above covariates, physical activity, fruit and vegetable intake, sleep, alcohol, and smoking status

None of the conceptualizations of depression, were associated with prostate cancer risk (**Table 5 and S4 Table**). Associations between anxiety, and comorbid anxiety and depression with prostate cancer are not presented due to low events (N = 9 and N = 5, respectively), but were similarly null.

**Table 5:** Hazard ratios between mental health disorders and subsequent diagnosis of prostate cancer.

	No. Events/No. of Participants	Person-years	Model 1† HR (95% CI)	Model 2‡ HR (95% CI)
**Depression (PHQ-9)**				
No	319/15178	94541	Ref	Ref
Yes	10/741	4725	0.74 (0.40–1.39)	0.72 (0.38–1.36)
**Depression (Antidepressant use)**				
No	311/14999	93808	Ref	Ref
Yes	18/920	5458	1.03 (0.64–1.66)	1.03 (0.64–1.66)
**Depression (PHQ-9, antidepressant use, or self-report of physician diagnosis)**				
No	263/12751	79085	Ref	Ref
Yes	66/3168	20181	1.03 (0.79–1.35)	1.03 (0.78–1.35)
**Anxiety (GAD-7)**				
No	320/15341	93808	Ref	Ref
Yes	9/578	5458	-	-
**Comorbid anxiety and depression**				
No	324/15580	97037	Ref	Ref
Yes	5/339	2230	-	-

- indicates suppression due to cell size <10

†Adjusted for age (continuous), education, and ethnicity

‡Adjusted for age (continuous), education, ethnicity, physical activity, fruit and vegetable consumption, and sleep

Depression—assessed by PHQ-9—was associated with an increased risk of lung cancer among women in Model 1: HR: 1,88 (95% CI 1.15–3.09), and Model 2: HR: 1.75 (95% CI 1.06–2,88) (**Table 6 and S5 Table**). Comorbid anxiety and depression were also associated with increased risk of lung cancer among women in Model 1; HR: 2.32 (95% CI: 1.28–4.20) and Model 2: HR: 2.11 (1.16–3.85). Additional adjustment for lifestyle variables in Model 3 attenuated these associations. Anxiety was associated with increased risk of lung cancer in women (**Table 6**). Associations remained with full adjustment for confounders (Model 3; HR: 1.67 (95% CI 1.01–2.76).

**Table 6:** Hazard ratios between mental health disorders and subsequent diagnosis of lung cancer, stratified by sex.

	No. of Events/No. of Participants	Person-years	Model 1† HR (95% CI)	Model 2‡ HR (95% CI)	Model 3§ HR (95% CI)
**Depression (PHQ-9)**					
Women					
No	137/17474	105482	Ref	Ref	Ref
Yes	18/1178	7348	**1.88 (1.15–3.09)**	**1.75 (1.06–2.88)**	1.44 (0.87–2.40)
Men					
No	142/15178	95143	Ref	Ref	Ref
Yes	8/741	4750	-	-	-
**Depression (Antidepressant use)**					
Women					
No	133/16381	99870	Ref	Ref	Ref
Yes	22/2271	12960	1.25 (0.79–1.96)	1.20 (0.76–1.88)	1.05 (0.67–1.67)
Men					
No	141/14999	94399	Ref	Ref	Ref
Yes	9/920	5494	-	-	-
**Depression (PHQ-9, antidepressant use, or self-report of physician diagnosis)**					
Women					
No	94/12677	76277	Ref	Ref	Ref
Yes	61/5975	36554	1.34 (0.97–1.86)	1.28 (0.92–1.77)	1.12 (0.81–1.56)
Men					
No	116/12751	79584	Ref	Ref	Ref
Yes	34/3168	20309	1.15 (0.79–1.69)	1.07 (0.72–1.57)	0.89 (0.60–1.31)
**Anxiety (GAD-7)**					
Women					
No	137/17641	99870	Ref	Ref	Ref
Yes	18/1011	12960	**2.04 (1.24–3.35)**	**1.91 (1.16–3.15)**	**1.67 (1.01–2.76)**
Men					
No	142/15341	94399	Ref	Ref	Ref
Yes	8/578	5494	-	-	-
**Comorbid anxiety and depression**					
Women					
No	143/18053	108955	Ref	Ref	Ref
Yes	12/599	3875	**2.32 (1.28–4.20)**	**2.11 (1.16–3.85)**	1.72 (0.94–3.16)
Men					
No	145/15580	97659	Ref	Ref	Ref
Yes	5/339	2234	-	-	-

- indicates suppression due to cell size <10

†Model 1: Adjusted for age (continuous), education, and ethnicity

‡Model 2: Adjusted for age (continuous), education, ethnicity, and COPD

**§**Model 3: Adjusted for all above covariates, physical activity, alcohol, fruit and vegetable consumption, and smoking status

### Mediation effect of lifestyle factors

The depression- or anxiety-cancer associations with a statistically significant direct effect are shown in **Table 7**. Smoking status was shown to partially mediate the relationship between depression (PHQ-9), anxiety, and co-morbid depression and anxiety on lung cancer in women by a proportion of 27%, 18%, and 26%, respectively. No interactions between exposures (depression, anxiety, and co-morbid depression) and mediators were observed among women, with the exception of smoking status.

**Table 7:** Mediation effects of lifestyle factors in the relationship between mental health and lung cancer in women and all cancer in men^a^^,^ ^b^.

Mediation effects		Quasi-Bayesian estimates and 95% CI			Estimated proportion mediated
Mediators	Associations	Indirect effect of X on Y	Direct effect of X on Y	Total effect (95% CI)	
**Women**					
Smoking status	Depression (PHQ-9) on lung cancer	0.00 (0.0009–0.0030) ***	0.01 (0.0004–0.0142) *	0.01 (0.0008–0.0153) *	0.27
	Anxiety on lung cancer	0.00 (0.0006–0.0026) ***	0.01 (0.0005–0.0167) *	0.01 (0.0013–0.0175) *	0.18
	Comorbidity on lung cancer	0.00 (0.0012–0.0046) ***	0.01 (0.0002–0.0211) *	0.01 (0.0017–0.0225) **	0.26
Alcohol consumption	Depression (PHQ-9) on lung cancer	0.00 (-0.0003–0.0008)	0.01 (0.0004–0.0150) *	0.01 (0.0006–0.0150) *	0.03
	Anxiety on lung cancer	0.00 (-0.0002–0.0006)	0.01 (0.0015–0.0169) *	0.01 (0.0017–0.0170) **	0.02
	Comorbidity on lung cancer	0.00 (-0.0003–0.0009)	0.01 (0.0016–0.0232) *	0.01 (0.0017–0.0232) **	0.03
BMI	Depression (PHQ-9) on lung cancer	-0.00 (-0.0015–0.0003)	0.01 (0.0009–0.0145) *	0.01 (0.0006–0.0144) *	-0.09
	Anxiety on lung cancer	-0.00 (-0.0010–0.0001)	0.01 (0.0022–0.0170) **	0.01 (0.0021–0.0169) **	-0.04
	Comorbidity on lung cancer	-0.00 (-0.0015–0.0003)	0.01 (0.0021–0.0232) *	0.01 (0.0018–0.0228) *	-0.05
Fruit and vegetable consumption	Depression (PHQ-9) on lung cancer	0.00 (-0.0003–0.0009)	0.01 (0.0004–0.0149) *	0.01 (0.0005–0.0149) *	0.03
	Anxiety on lung cancer	0.00 (-0.0003–0.0009)	0.01 (0.0017–0.0174) **	0.01 (0.0019–0.0175) **	0.02
	Comorbidity on lung cancer	0.00 (-0.0004–0.0011)	0.01 (0.0017–0.0220) *	0.01 (0.0019–0.0222) *	0.03
Sleep	Depression (PHQ-9) on lung cancer	-0.00 (-0.0008–0.0002)	0.01 (0.0014–0.0170) **	0.01 (0.0014–0.0168) **	-0.03
	Anxiety on lung cancer	-0.00 (-0.0007–0.0002)	0.01 (0.0019–0.0167) **	0.01 (0.0018–0.0166) **	-0.03
	Comorbidity on lung cancer	-0.00 (-0.0011–0.0003)	0.03 (0.0015–0.0229) *	0.01 (0.0014–0.0227) *	-0.03
**Men**					
Smoking status	Depression^§^ on all cancer	0.00 (0.0011–0.0039) ***	0.01 (0.0003–0.0229) *	0.01 (0.0021–0.0247) *	0.17
Alcohol consumption	Depression^§^ on all cancer	0.00 (-0.0002–0.0003)	0.01 (0.0027–0.0252) *	0.01 (0.0027–0.0252) *	0.00
BMI	Depression^§^ on all cancer	0.00 (-0.0004–0.0005)	0.01 (0.0017–0.0247) *	0.01 (0.0018–0.0250) *	0.00
Fruit and vegetable intake	Depression^§^ on all cancer	0.0000 (-0.0002–0.0002)	0.0134 (0.0026–0.0252) *	0.0134 (0.0026–0.0253) *	-0.00
Sleep	Depression^§^ on all cancer	-0.0002 (-0.0007–0.0003)	0.0125 (0.0019–0.0234) *	0.0124 (0.0019–0.0232) *	-0.01

*p ≤ 0.05

**p≤ 0.01

***p ≤ 0.001

^a^ All models were adjusted for age, sex, education, ethnicity, income, and family history of cancer

^b^ quasi-Bayesian approximation based on 1000 simulations

†Anxiety defined as GAD-7 scores over or equal to 10

‡Co-morbidity defined as both GAD-7 and PHQ-9 scores over or equal to 10

^§^Defined by a positive result for the PHQ-9, antidepressant use, or lifetime self-report of physician diagnosis

Mediation effects for men were examined for only depression and all cancer as this was the only relationship that was statistically significant. Smoking status was shown to partially mediate the relationship between depression (PHQ-9, antidepressant use, or self-report of lifetime physician diagnosis) on all cancer in men by a proportion of 17%. No interactions between depression and any mediator were observed among men.

## Discussion

Findings from this large study of Canadians from Quebec suggest that there may be a modest positive association between mental health disorders and risk of all cancers and lung cancer. These relationships persisted with adjustment for sociodemographic and health variables but were generally attenuated with adjustment for lifestyle factors, showing both the importance of adjustment for these factors in future studies and further underscoring the importance of healthy lifestyles for cancer prevention. Further, smoking may be an important and modifiable health behaviour that contributes in part to relationships between depression (PHQ-9), anxiety (GAD-7), and co-morbid depression and anxiety and lung cancer risk in women. Similarly in men, findings suggest that of the lifestyle variables explored in our study, smoking may be the largest contributor to relationships between depression (PHQ-9, antidepressant use, or lifetime self-report of physician diagnosis) and all cancer incidence.

Evidence on the relationship between mental health disorders and cancer risk is inconsistent; with some studies reporting significant positive relationships for some cancer subtypes but not others, and other studies presenting non-significant findings [8, 9, 50, 51]. Differences between our research and previous studies might reflect the differences in international screening practices, different lifestyle factors and assessment of lifestyle factors, varying methods of ascertaining cancer status (e.g., self-report and cancer registry) and mental health status (e.g., DSM criteria, questionnaire scores, and administrative health databases). For example, Gross et al. conducted a longitudinal study with up to 24 years of follow-up, and found that there was a significant positive association between depression and all cancer risk (HR: 1.9, 95% CI: 1.2, 3.0) [50]. However, they did not adjust for any health behaviours other than smoking, which may obscure true associations due to confounding [50]. Consistent with our results, O’Neill et al. found no significant association between depression and all cancer risk, but also found that risk estimates were higher in women than men [51].

The present study primarily focussed on mental health exposures as presence or absence which may not capture the range of symptoms or specific aspects of mental health that are relevant for cancer. A cohort study in Korea found inconsistent results within their research on mental health disorders and prostate cancer risk. In a dose-response analysis, mild depression was significantly associated with subsequent prostate cancer risk, while moderate and severe depression were not [52]. Interestingly, in a separate assessment of minor and major depression, only minor depression was significantly associated with increased prostate cancer risk [52]. These studies may suggest that mild forms of depression, which would not be captured in our study, may be associated with prostate cancer risk. However, our additional analyses which examined PHQ-9 scores and GAD-7 scores on a continuous scale were consistent with the main analyses of presence/absence of anxiety and depression.

The results of our mediation analysis are consistent with the findings of Trudel-Fitzgerald et al., who found that lifetime pack-years of smoking mediated 38% of the overall association between depressive symptoms and lung cancer risk in women [53]. Since smoking is the predominant risk factor for lung cancer [54, 55] this finding is biologically plausible. To our knowledge, there is no comparable study that assessed this relationship in a cohort with men. A study by Burns et al. assessed the relationship between mental health difficulties, which was defined as any emotional, nervous, or psychiatric problems, and smoking-related diseases, which included respiratory disease, cardiovascular disease, or any smoking-related cancer [56]. They found that smoking had no significant mediating role in the relationship between mental health difficulties and smoking-related diseases [56]. However, this study grouped different mental health difficulties and smoking-related diseases together as a single exposure and outcome, limiting its comparability [56]. Given the limited number of studies that have assessed this relationship, additional research is warranted. Future studies that consider a wider range of potential mediators may help to provide insight on mental health-cancer links since smoking explained less than a third of the associations in this study.

A strength of this study was the large cohort size that included men and women reporting a wide range of income and education, which increases the generalizability of the findings [27]. A further strength was the collection of information on mental health disorders at baseline using standard validated questionnaires, along with detailed information about health behaviours using self-reported and measured data (BMI). Previous studies have seldom considered health behaviours as potential confounders in analyses. The availability of multiple variables related to depression at baseline allowed for conceptualizations of depression that may capture different dimensions or chronicity of depression. For example, anti-depressive medication use may reflect more severe or longer depressive symptoms versus the PHQ-9 which reflects depressive symptoms in the prior two weeks. The general consistency of findings across different depression definitions increases the confidence in our results.

Limitations of this study include the short overall follow-up time, which given the long latency of most cancers may not have accurately captured the association between mental health disorders and cancer incidence. The short follow-up was also reflected in a modest number of incident cancers. This may have obscured more modest associations between mental health and cancer risk in the analyses of cancer sites and further stratification by sex. Although individuals with an incident cancer in the first six months of follow-up were excluded, it is possible that participants had asymptomatic or early stages of cancer that were not captured at baseline. Another limitation was the limited availability on incidence of cancer sites beyond prostate and lung cancer due to restrictions on data availability at the time of our analysis. Mental health variables and lifestyle factors were measured simultaneously at baseline within the CARTaGENE cohort. Assessment of exposure and mediator variables at the same time point weakens the argument for causation in the mediation analysis, which limits the robustness of findings from this part of the study [57]. Caution is also warranted in the interpretation of direct and indirect effects of smoking in the mediation analysis in women, given the presence of an exposure-mediator interaction [49].

Additional studies which consider cancers that are strongly associated with lifestyle factors such as breast and colorectal cancer [54, 58] may help further elucidate potential mediators of mental health disorders and cancer risk. Other cancer outcomes, such as mortality and survival, are also of interest for future research. Similar to cancer incidence, there is a reasonable body of evidence suggesting a positive association between mental health disorders and cancer outcomes [59, 60]. Based on observations from previous literature and results from the present study, future research investigating this relationship should aim to stratify by sex, assess lifestyle behaviours, and explore alternative conceptualizations of mental health.

## Conclusion

This study demonstrated a modest positive association between mental health disorders and lung cancer risk in women, however only anxiety and lung cancer in women remained significant with full adjustment of sociodemographic, health and lifestyle factors. The mediation analysis found that smoking may contribute to associations between anxiety, co-morbid depression and anxiety, and depression with incident lung cancer in women, and associations between depression with all cancers in men. This provides further emphasizes the importance of ongoing public health efforts to reduce smoking.

## Supporting information

S1 File Supporting information contains all the S1-S7 Tables. (DOCX)
