# Supplementary material for: Mental health, cancer risk, and the mediating role of lifestyle factors in the CARTaGENE cohort study
Source: PLoS One. 2023 Feb 14;18(2):e0281588. doi: 10.1371/journal.pone.0281588 (PMC9928103; doi:10.1371/journal.pone.0281588)
Supplement: S1 File — (DOCX) [file pone.0281588.s001.docx]

**Table S1**. Hazard ratios between mental health disorders (continuous scores) and subsequent diagnosis of all cancers, stratified by sex

|  | **No. of Events/No. of Participants** | **Person-years** | **Model 1**†  HR (95% CI) | **Model 2**‡  HR (95% CI) | **Model 3§**  HR (95% CI) |
| --- | --- | --- | --- | --- | --- |
| **Depression (PHQ-9) Continuous Scores** |  |  |  |  |  |
| Women | 1527/18652 | 108505 | 1.01 (1.00-1.02) | 1.01 (0.99-1.02) | 1.00 (0.99-1.02) |
| Men | 1361/15919 | 95882 | 1.01 (1.00-1.03) | 1.01 (1.00-1.03) | 1.01 (0.99-1.02) |
| **Anxiety (GAD-7) Continuous Scores** |  |  |  |  |  |
| Women | 1527/18652 | 108505 | 1.01 (0.99-1.02) | 1.01 (0.99-1.02) | 1.00 (0.99-1.02) |
| Men | 1361/15919 | 95882 | 1.00 (0.98-1.02) | 1.00 (0.99-1.02) | 1.00 (0.98-1.02) |

†Model 1: Adjusted for age (continuous), education, ethnicity, and income

‡Model 2: Adjusted for age (continuous), education, ethnicity, income, myocardial infarction, diabetes, and family history of cancer

**§**Model 3: Adjusted for all above covariates, physical activity, fruit and vegetable intake, sleep, alcohol, and smoking status

**Table S2**. Hazard ratios between mental health disorders (continuous scores) and subsequent diagnosis of all cancers for men and women combined

|  | **No. of Events/No. of Participants** | **Person-years** | **Model 1**†  HR (95% CI) | **Model 2**‡  HR (95% CI) | **Model 3§**  HR (95% CI) |
| --- | --- | --- | --- | --- | --- |
| **Depression (PHQ-9) Continuous Scores** | 2888/34571 | 204387 | 1.01 (1.00-1.02) | 1.01 (1.00-1.02) | 1.00 (0.99-1.01) |
| **Anxiety (GAD-7) Continuous Scores** | 2888/34571 | 204387 | 1.00 (0.99-1.02) | 1.00 (0.99-1.01) | 1.00 (0.99-1.01) |

†Model 1: Adjusted for age (continuous), education, ethnicity, and income

‡Model 2: Adjusted for age (continuous), education, ethnicity, income, myocardial infarction, diabetes, and family history of cancer

**§**Model 3: Adjusted for all above covariates, physical activity, fruit and vegetable intake, sleep, alcohol, and smoking status

**Table S3**. Hazard ratios between mental health disorders and subsequent diagnosis of all cancers for men and women combined

|  | **No. of Events/No. of Participants** | **Person-years** | **Model 1**†  HR (95% CI) | **Model 2**‡  HR (95% CI) | **Model 3§**  HR (95% CI) |
| --- | --- | --- | --- | --- | --- |
| **Depression (PHQ-9)** |  |  |  |  |  |
| No | 2720/32652 | 192772 | – | – | – |
| Yes | 168/1919 | 11615 | 1.11 (0.95-1.30) | 1.11(0.95-1.30) | 0.99 (0.85-1.44) |
| **Depression (Antidepressant use)** |  |  |  |  |  |
| No | 2626/31380 | 186610 | – | – | – |
| Yes | 262/3191 | 17777 | 1.06 (0.93-1.20) | 1.05 (0.93-1.20) | 1.01 (0.89-1.15) |
| **Depression (PHQ-9, antidepressant use, or self-report of physician diagnosis)** |  |  |  |  |  |
| No | 2068/25428 | 149859 | – | – | – |
| Yes | 816/9143 | 54526 | **1.09 (1.01-1.19)** | 1.09 (1.00-1.18) | 1.11 (0.93-1.32) |
| **Anxiety (GAD-7)** |  |  |  |  |  |
| No | 2744/32982 | 186610 | – | – | – |
| Yes | 144/1589 | 17777 | 1.10 (0.93-1.30) | 1.10 (0.93-1.30) | 0.91 (0.67-1.24) |
| **Comorbid anxiety and depression** |  |  |  |  |  |
| No | 2794/33633 | 198543 | – | – | – |
| Yes | 94/938 | 5844 | **1.25 (1.02-1.54)** | **1.24 (1.01--1.53)** | 1.17 (0.95-1.44) |

†Model 1: Adjusted for age (continuous), education, ethnicity, and income

‡Model 2: Adjusted for age (continuous), education, ethnicity, income, myocardial infarction, diabetes, and family history of cancer

**§**Model 3: Adjusted for all above covariates, physical activity, fruit and vegetable intake, sleep, alcohol, and smoking status

**Table S4**. Hazard ratios between mental health disorders (continuous scores) and subsequent diagnosis of prostate cancer

|  | **No. of Events/No. of Participants** | **Person-years** | **Model 1**†  HR (95% CI) | **Model 2**‡  HR (95% CI) |
| --- | --- | --- | --- | --- |
| **Depression (PHQ-9) Continuous Scores** | 329/15919 | 99266 | 1.00 (0.97-1.03) | 1.00 (0.96-1.03) |
| **Anxiety (GAD-7) Continuous Scores** | 329/15919 | 99266 | 1.00 (0.97-1.04) | 1.00 (0.96-1.04) |

†Adjusted for age (continuous), education, and ethnicity

‡Adjusted for age (continuous), education, ethnicity, physical activity, fruit and vegetable consumption, and sleep

**Table S5**. Hazard ratios between mental health disorders (continuous scores) and subsequent diagnosis of lung cancer, stratified by sex

|  | **No. of Events/No. of Participants** | **Person-years** | **Model 1**†  HR (95% CI) | **Model 2**‡  HR (95% CI) | **Model 3§**  HR (95% CI) |
| --- | --- | --- | --- | --- | --- |
| **Depression (PHQ-9) Continuous Scores** |  |  |  |  |  |
| Women | 155/18652 | 112830 | **1.05 (1.01-1.09)** | **1.04 (1.01-1.08)** | 1.03 (0.99-1.06) |
| Men | 150/15919 | 99893 | 1.03 (0.98-1.07) | 1.02 (0.97-1.06) | 0.99 (0.95-1.04) |
| **Anxiety (GAD-7) Continuous Scores** |  |  |  |  |  |
| Women | 155/18652 | 112830 | **1.05 (1.01-1.08)** | **1.04 (1.00-1.08)** | 1.03 (0.99-1.07) |
| Men | 150/15919 | 99893 | 1.02 (0.98-1.07) | 1.02 (0.97-1.06) | 1.00 (0.95-1.05) |

†Model 1: Adjusted for age (continuous), education, and ethnicity

‡Model 2: Adjusted for age (continuous), education, ethnicity, and health status (COPD)

**§**Model 3: Adjusted for all above covariates, physical activity, alcohol, fruit and vegetable consumption, and smoking status

**Table S6**. Hazard ratios between mental health disorders (continuous scores) and subsequent diagnosis of lung cancer for men and women combined

|  | **No. of Events/No. of Participants** | **Person-years** | **Model 1**†  HR (95% CI) | **Model 2**‡  HR (95% CI) | **Model 3§**  HR (95% CI) |
| --- | --- | --- | --- | --- | --- |
| **Depression (PHQ-9) Continuous Scores** | 305/34571 | 212723 | **1.04 (1.01-1.07)** | 1.03 (1.00-1.06) | 1.01 (0.99-1.04) |
| **Anxiety (GAD-7) Continuous Scores** | 305/34571 | 212723 | **1.04 (1.01-1.07)** | 1.03 (1.00-1.06) | 1.02 (0.99-1.05) |

†Model 1: Adjusted for age (continuous), education, and ethnicity

‡Model 2: Adjusted for age (continuous), education, ethnicity, and health status (COPD)

**§**Model 3: Adjusted for all above covariates, physical activity, alcohol, fruit and vegetable consumption, and smoking status

**Table S7**. Hazard ratios between mental health disorders and subsequent diagnosis of lung cancer for men and women combined

|  | **No. of Events/No. of Participants** | **Person-years** | **Model 1**†  HR (95% CI) | **Model 2**‡  HR (95% CI) | **Model 3§**  HR (95% CI) |
| --- | --- | --- | --- | --- | --- |
| **Depression (PHQ-9)** |  |  |  |  |  |
| No | 279/32652 | 200625 | – | – | – |
| Yes | 26/1919 | 12068 | 1.62 (1.08-2.43) | 1.47 (0.98-2.22) | 1.20 (0.79-1.81) |
| **Depression (Antidepressant use)** |  |  |  |  |  |
| No | 274/31380 | 194269 | – | – | – |
| Yes | 31/3191 | 18454 | 1.21 (0.83-1.75) | 1.14 (0.79-1.66) | 1.00 (0.68-1.45) |
| **Depression (PHQ-9, antidepressant use, or self-report of physician diagnosis)** |  |  |  |  |  |
| No | 210/25428 | 155861 | – | – | – |
| Yes | 95/9143 | 56863 | 1.24 (0.97-1.58) | 1.17 (0.91-1.49) | 1.01 (0.78-1.29) |
| **Anxiety (GAD-7)** |  |  |  |  |  |
| No | 279/32982 | 194269 | – | – | – |
| Yes | 26/1589 | 18454 | **1.83 (1.22-2.75)** | **1.70 (1.13-2.55)** | 1.45 (0.96-2.19) |
| **Comorbid anxiety and depression** |  |  |  |  |  |
| No | 288/33633 | 206614 | – | – | – |
| Yes | 17/938 | 6109 | **2.08 (1.27-3.41)** | **1.87 (1.14-3.08)** | 1.51 (0.92-2.49) |

†Model 1: Adjusted for age (continuous), education, and ethnicity

‡Model 2: Adjusted for age (continuous), education, ethnicity, and health status (COPD)

**§**Model 3: Adjusted for all above covariates, physical activity, alcohol, fruit and vegetable consumption, and smoking status
